# Survival and prognostic factors for survival, cancer specific survival and disease free interval in 239 patients with Hurthle cell carcinoma: a single center experience

**DOI:** 10.1186/s12885-017-3370-x

**Published:** 2017-05-25

**Authors:** Branisav Oluic, Ivan Paunovic, Zlatibor Loncar, Vladimir Djukic, Aleksandar Diklic, Milan Jovanovic, Zeljko Garabinovic, Nikola Slijepcevic, Branislav Rovcanin, Dusan Micic, Aleksandar Filipovic, Vladan Zivaljevic

**Affiliations:** 1 Emergency Center, Clinical Center of Serbia, Pasterova 2, Belgrade, 11000 Serbia; 2 Center for Endocrine Surgery, Clinical Center of Serbia, Pasterova 2, Belgrade, 11000 Serbia; 3 Faculty of Medicine, University of Belgrade, Dr Subotica 8, Belgrade, 11000 Serbia; 4 Clinic for Thoracic Surgery, Clinical Center of Serbia, Pasterova 2, Belgrade, 11000 Serbia; 5 Clinical Center of Montenegro, Department of Endocrine Surgery, University of Montenegro, Podgorica, Montenegro

**Keywords:** Thyroid gland, Hurthle cell carcinoma, Survival, Cancer specific survival, Disease free interval

## Abstract

**Background:**

Hurthle cell carcinoma makes up 3 to 5% of all thyroid cancers and is considered to be a true rarity. The aim of our study was to analyze clinical characteristics and survival rates of patients with Hurthle cell carcinoma.

**Methods:**

Clinical data regarding basic demographic characteristics, tumor grade, type of surgical treatment and vital status were collected. Methods of descriptive statistics and Kaplan-Meier survival curves were used for statistical analysis. Cox proportional hazards regression was used to identify independent predictors.

**Results:**

During the period from 1995 to 2014, 239 patients with Hurthle cell carcinoma were treated at our Institution. The average age of the patients was 54.3, with female to male ratio of 3.6:1 and average tumor size was 41.8 mm. The overall recurrence rate was 12.1%, with average time for relapse of 90.74 months and average time without any signs of the disease of 222.4 months. Overall 5-year, 10-year and 20-year survival rates were 89.4%, 77.2%, 61.9% respectively. The 5-year, 10-year and 20-year cancer specific survival rates were 94.6%, 92.5%, 87.4%, respectively. When disease free interval was observed, 5-year, 10-year and 20-year rates were 91.1%, 86.2%, 68.5%, respectively. The affection of both thyroid lobes and the need for reoperation due to local relapse were unfavorable independent prognostic factors, while total thyroidectomy as primary procedure was favorable predictive factor for cancer specific survival.

**Conclusion:**

Hurthle cell carcinoma is a rare tumor with an encouraging prognosis and after adequate surgical treatment recurrences are rare.

## Background

Thyroid gland cancers are relatively rare tumors which represent approximately 1% of all malignant tumors, and in the structure of all malignant tumors-related lethal outcomes, they are accounted for less than 0.5%. Hurthle cell carcinoma or oxyphilic carcinoma makes up 3 to 5% of all thyroid cancers and, therefore, is considered to be a true rarity [1, 2].

According to the current classification of the World Health Organization, Hurthle cell carcinoma represents a variant of follicular carcinoma of the thyroid gland. Still, genetic studies have shown that these tumors have a completely different oncogenesis; furthermore, there are differences regarding clinical characteristics if compared to papillary and follicular carcinoma [3]. In order to be classified as a Hurthle cell carcinoma, a tumor should predominantly consist of the Hurthle cells, which originate from follicular epithelium of the thyroid gland.

As for all other thyroid cancers, surgery represents primary and basic treatment method for Hurthle cell carcinoma, as well. When it comes to radioiodine treatment for this tumor, there are still ongoing debates, since it bonds radioactive iodine slightly lower compared to other well-differentiated thyroid cancers.

In the past, this type of thyroid cancer was associated with prognosis which was worse than the prognosis of papillary and follicular cancers [1, 4].

Given the low incidence rate, studies which have examined the clinical characteristics and optimal treatment methods of Hurthle cell carcinoma are rare and mostly based on individual institution’s experience. When it comes to survival rates, cancer specific survival rates, disease free interval, as well as prognostic factors for this tumor, literature is particularly deficient.

The aim of our paper was to analyze the basic clinical and pathological characteristics of this tumor and to determine the overall survival, cancer-specific survival and disease free interval in patients with Hurthle cell carcinoma on a representative number of subjects. The second aim was to determine and analyze prognostic factors and predictors of survival in patients with Hurthle cell carcinoma.

## Methods

A retrospective cohort study was conducted at our Institution, from January 1st 1995 until December 31st 2014. The study included all patients in whom the diagnosis of oxyphilic thyroid cancers was confirmed by a definitive histopathological examination. All preparations were re-examined by a pathologist, experienced in the field of endocrine surgery, so the diagnosis of Hurthle cell carcinoma was confirmed.

Based on the insight into medical documentation (medical history and an electronic database implemented in everyday work) we have collected data of all patients regarding the following: basic demographic characteristics of patients (gender, age), duration of disease, familial form of cancer, clinical features (TNM (Tumor Nodes Metastasis) classification of tumors, associated thyroid gland diseases, thyroid hormone levels, antibodies and markers’ levels, regional lymph-node metastasis, distant metastasis, infiltration of adjacent organs), histopathologic characteristics of the tumor, surgical treatment (type of surgery, the year of the procedure, the experience of the surgeon, reoperation due to local relapse), other treatment forms (radioiodine therapy, radiotherapy, substitutional-suppressive levothyroxine therapy) and associated diseases (diabetes, other malignant tumors).

**Fig. 1:**
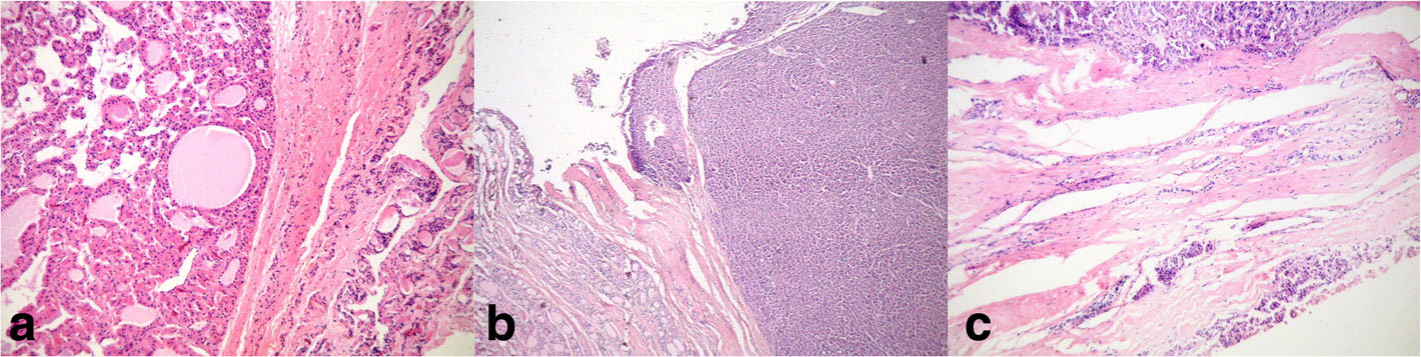
Hurthle cell carcinoma: **a**) without capsular or vascular invasion, **b**) capsular invasion, **c**) vascular invasion

The duration of illness was defined as the period from the time of diagnosis of thyroid gland changes until the time of surgery. Familial form of the disease was defined if patients had the first degree relatives who have undergone surgery due to Hurthle cell carcinoma. Staging of the tumor was carried out on the basis of TNM classification, which was valid at the time of operation, based on the histopathological findings. Data on the size of the tumor were collected separately, so no reclassification was done due to changes in criteria in 2002 [5]. Associated thyroid diseases were determined based on preoperative or histopathological findings. Serum levels of thyroid hormones, antibodies and markers (thyroid-stimulating hormone, triiodothyronine, thyroxine, thyroperoxidase antibody and thyroglobulin) were measured just before the surgery. All patients were euthyroid at the time of surgery. Patients with hyperthyroidism were treated until the euthyroid state was achieved and subsequently treated surgically. Data regarding regional lymph node metastases and distant metastases at the time of operation were also collected. Infiltration of the surrounding tissue was determined based on intraoperative and histopathological findings. Characteristics of the tumor were determined based on histopathological findings: invasive or minimally invasive type of tumor, tumor size, capsule invasion, vascular invasion, multicentricity, involvement of one or both lobes (Fig. 1).

Data regarding patients’ survival/death (whether patients were still alive and if not when they died) were obtained through the contact with patients or members of their families. We have also collected data of local recurrences of the tumor or metastases and further treatment methods. All patients received substitutional-suppressive levothyroxine therapy in order to prevent recurrence of the tumor.

Until 2006, radioiodine was applied only in cases of advanced tumors and later on in all cases in which total thyroidectomy was performed, where tumor was over T1.

We have particularly analyzed surgical treatment of Hurthle cell carcinomas. Patients were classified into groups according to the type of operation: total thyroidectomy (total thyroidectomy or near total thyroidectomy) and hemithyroidectomy group (lobectomy or near lobectomy). Whenever enlarged lymph nodes were present (seen by an ultrasound examination or intraoperatively) appropriate dissection was done. In patients in whom lobectomy was performed, we have searched the data regarding the completion of thyroidectomy. Completion of total thyroidectomy following lobectomy was done when the contralateral side was altered or when there was clearly unilateral invasive cancer, so ablative radioiodine therapy was indicated. When patients were re-operated following total thyroidectomy due to local relapse, or when the focus of the tumor was found during the completion of total thyroidectomy following lobectomy, we have marked that those patients had recurrence of the cancer.

### Statistical analysis

Statistical analyses were performed using SPSS 22.0 software (SPSS Inc., Chicago, IL).

The quantitative variables were expressed as mean ± standard deviation (SD), while the categorical ones were presented as percentages. Kaplan-Meier survival curves were used for determination of overall survival, cancer specific survival and disease free interval for each observed variable. The log rank test was used to determine the overall survival rate, cancer specific survival rate and disease-free survival interval. At last, univariate Cox regression analysis was performed in order to determine which variables were significantly associated with survival. Those variables that have showed significant association with survival in univariate analysis, at the level of significance of *p* ≤ 0.05, were included in multivariate analysis.

## Results

During 20-years period covered by the study, 13.385 patients were surgically treated due to thyroid gland diseases at our highly specialized center for endocrine surgery. Out of this number, a total of 3.344 thyroid cancers (29.98%) were histopathologically verified. Hurthle cell carcinoma was present in 239 patients (diagnosed by histopathologic examinations), which represents 7.1% of all thyroid cancers and 1.8% of all patients who have undergone thyroid gland surgery.

Table 1 presents demographic characteristics of patients and basic characteristics of carcinomas. The average age of patients with Hurthle cell carcinoma at the time of surgery was 54.3 ± 13.7 years; the youngest patient was 20 and the oldest 89 years old. The vast majority of affected individuals were in the 6th decade of their life. Females were more affected and female to male ratio was 3.6:1.

**Table 1:** Demographic and clinical characteristics of patients with Hurthle cell carcinoma

Variable	Number	Percent
Gender		
Female	187	78.2
Males	52	21,8
Age	54,3 ± 13,7	
20–29	10	4,2
30–39	28	11,7
40–49	46	19,2
50–59	66	27,6
60–69	53	22,2
> 70	36	15,1
pT tumor stage		
T1	19	7,9
T2	112	46,9
T3	75	31,4
T4	33	13,8
N stage		
N0	72	30,1
N1a	5	2,1
N1b	1	0,4
Nx	161	67,4
Tumor invasiveness		
minimally invasive	102	67,1
widely invasive	50	32,9
Tumor size (mm)		
<39	93	46
≥40	109	54
Capsular invasion		
transcapsular	152	69,7
no or minimal	66	30,3
Vascular invasion		
positive	146	67
no or minimal	72	33
Number of tumor focuses		
one focus	167	76,6
multicentric	51	21,3
Regional tumor infiltration		
positive	33	13,8
negative	206	86,2
Thyroglobulin		
normal values (<68 ng/mL)	19	26,8
elevated values (>68 ng/mL)	52	73,2
Radioiodine ablation after surgery		
Yes	65	36,9
No	111	63,1
Surgeon experience		
resident	11	4,6
specialist 1–5 years	20	8,4
specialist 6–10 years	57	23,8
specialist over 10 years	151	63,2

The average duration of the disease, from the moment of diagnosis of the alteration in the thyroid gland up to surgery was 77 months. In two patients (0.8%) there was a positive family history of thyroid oxyphilic carcinoma (in one case, the patient’s father and in the other, the patient’s mother).

The median tumor size in surgically treated patients due to Hurthle cell carcinoma was 41.8 mm (range 4 mm - 160 mm). Most of the tumors were classified as minimally invasive, in 102 patients (67.1%). Invasion of the capsule was present in 152 patients (69.7%) and vascular invasion in 146 patients (67%). Tumor usually had one focus, in 167 patients (76.6%). In the group of patients in whom bilateral procedure was performed (162 patients), the tumor was unilateral in 116 patients (71.6%). The tumor was most often in the T2 stage (112 patients, 46.9%) (Table 1).

Preoperative values of thyroglobulin were elevated in 52 patients (73.2%). Regional lymph nodes metastases at the time of surgery existed in 6 patients (2.8%), while the local infiltration of surrounding tissue was present in 33 patients (13.8%). In 7.7% of the patients whose lymph nodes were extirpated during the primary surgery, lymphatic metastases were present. Twelve patients (5%) also had micropapillary carcinoma; in 3 patients, oxyphilic carcinoma was developed focally within benign lesions: 2 in follicular adenoma and one in colloid adenoma (Table 2). Another malignancy existed in 19 patients (8%) and the most common localization of the other tumor was genitourinary system (7 patients, 36.9%).

**Table 2:** Primary procedure and associated thyroid gland diseases in patients with Hurthle cell carcinoma

Type of surgery	Number	Percent
Total thyroidectomy	160	66.9
Lobectomy	66^a^	27.6
Dunhill’s procedure	10	4.2
Tumor reduction	3	1.3
Associated thyroid pathology		
Micropapillary thyroid carcinoma	12	5.0
Papillary thyroid carcinoma	4	1.7
Medullary carcinoma	1	0.4
Graves’ disease	2	0.8
Thyroiditis	18	7.5
Benign tumor	3	1.3
Total	40	16.7

^a^ in 21 patients total thyroidectomy was performed after lobectomy

### Surgical procedures

In patients surgically treated for Hurthle cell carcinoma, the most commonly performed primary procedure was total thyroidectomy, in 160 patients (66.9%), while in 66 patients (27.6%) lobectomy was done (Table 2). Completion of thyroidectomy following lobectomy was performed in 21 patients, which represent 31.8% of all lobectomies. In 12 of these patients, focus of oxyphilic carcinoma was found on the contralateral side, accounting for 57.1% of all patients in whom completion of thyroidectomy was performed, or 18.2% of all patients in whom lobectomy was done. Completion was performed after an average of 14.4 ± 13.5 months (range, 3–36 months). In 78 patients central or lateral neck dissection was performed during the initial procedure and metastases in the lymph nodes were present in 6 patients (7.7%). The average length of surgeons’ specialist experience was 16.3 ± 9.8 years (range, 0–35 years) (Table 1). Radioiodine was applied in 65 patients (36.9%), most commonly once (in 58 patients).

### Outcomes

**Fig. 2:**
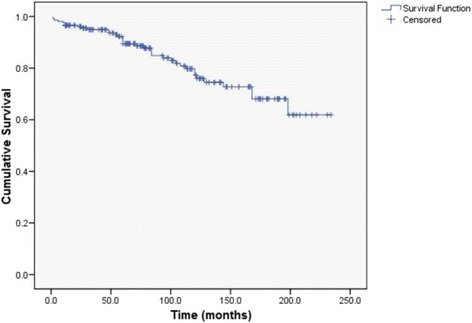
Kaplan-Meier overall survival curve

At the time of completion of the study, with an average follow-up period of 89.5 ± 60.2 months (range 1–234), 36 patients have died. Minimum follow-up period was one year (except for those patients who have died earlier). Thyroid gland disease was the cause of death in 13 patients (6.4%), i.e. in 36.1% of all deceased patients. At the moment of death, the average age of patients with Hurthle cell carcinoma who died because of thyroid pathology was 64.7 ± 12.1 years. On the other hand, average age at the moment of death of patients who were operated for Hurthle cell carcinoma and died due to other reasons was 74.4 ± 9.2 years. Average overall survival was 186.6 months (95% Confidence interval (CI): 173.3–199.9). An overall one-year survival rate was 96.6%, five-year survival was 89.4%, ten-year was 77.2% and twenty-year survival rate was 61.9%. (Fig. 2).

Univariate analysis for overall survival revealed that the following 6 factors were associated with shorter survival rate: age, T stadium, tumor type, tumor size, regional tumor infiltration, affection of both lobes, re-operation due to relapse and hypertension (Table 3). Multivariate regression analysis showed that independent unfavorable prognostic factors for overall survival were age over 55 (odds ratio (OR) 5.92, 95% CI 1.95–17.96), T3 and T4 tumor stadium (OR 4.31, 95% CI 1.67–11.09), affection of both lobes (OR 3.29, 95% CI 1.42–7.62) and re-operation due to relapse (OR 0.3, 95% CI 0.11–0.79).

**Table 3:** Univariate and multivariate statistical analysis of overall survival

	Univariate			Multivariate		
Factor	*p*	OR	95% CI	*p*	OR	95% CI
Gender (*male* vs. *female*)	0,281	0,67	0,32–1,39			
Age (years) (*<54* vs. *≥55*)	0,001	8,92	3,63–21,92	0,002	5,92	1,95–17,96
Disease duration (years) (*<5* vs. *≥5*)	0,421	1,31	0,68–2,53			
T stadium (*T1 and T2* vs. *T3 and T4*)	0,001	4,47	2,03–9,82	0,002	4,31	1,67–11,09
N stadium (*N0 and Nx* vs. *N1a and N1b*)	0,601	1,46	0,35–6,11			
Tumor type (*minimally* vs. *widely invasive*)	0,044	1,96	1,01–3,79			
Tumor size (*mm*) (*≤39* vs. *>40*)	0,004	4,19	1,57–11,19			
Capsular invasion (*positive* vs. *no or minimal*)	0,38	1,42	0,65–3,11			
Vascular invasion (*positive* vs. *no or minimal*)	0,287	0,61	0,25–1,50			
Number of tumor focuses (*one* vs. *multicentric*)	0,104	1,84	0,88–3,85			
Affection of both lobes (*yes* vs. *no*)	0,004	2,93	1,4–6,12	0,005	3,29	1,42–7,62
Regional tumor infiltration (*yes* vs. *no*)	0,003	0,35	0,17–0,7			
Primary procedure (*total thyroidectomy* vs. *less than total*)	0,65	1,18	0,56–2,48			
Total thyroidectomy after lobectomy (*yes* vs. *no*)	0,19	0,44	0,12–1,53			
Type of procedure overall (*total thyroidectomy* vs. *less than total*)	0,836	0,92	0,4–2,09			
Reoperation due to relapse (*yes* vs. *no*)	0,035	0,407	0,17–0,94	0,016	0,3	0,11–0,79
Surgeon’s experience (*years*) (*<10* vs. *10+*)	0,25	0,66	0,32–1,33			
Radioiodine ablation (*yes* vs. *no*)	0,43	1,88	0,39–9,08			
Diabetes mellitus (*yes* vs. *no*)	0,3	0,53	0,16–1,77			
Hypertension (*yes* vs. *no*)	0,002	0,32	0,16–0,65			
Other malignancies (*yes* vs. *no*)	0,275	0,55	0,19–1,59			
Thyroglobulin (ng/mL) (*<300* vs. *>300*)	0,925	1,11	0,11–10,9			

**Fig. 3:**
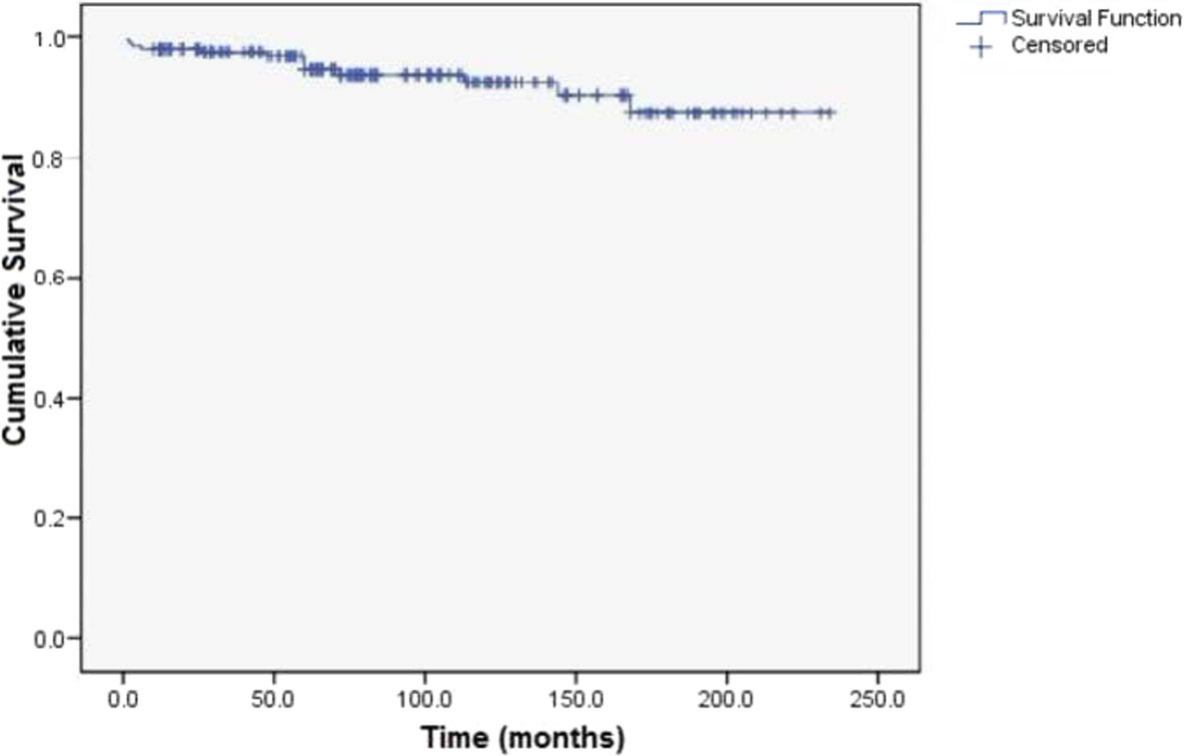
Kaplan-Meier cancer specific survival curve

Average cancer specific survival was 216.4 months (95% CI: 207.1 to 225.7). Cancer specific survival rate after one year was 98%, after five years was 94.6%, after ten years was 92.5% and twenty-year survival rate was 87.4%. (Fig. 3).

The results of univariate analysis for cancer specific survival showed that age, T stadium, regional tumor infiltration, affection of both lobes, primary operation and reoperation due to relapse contribute to shorter survival (Table 4). The results of multivariate regression analysis revealed that independent prognostic factors for cancer specific survival were affection of both lobes (OR 8.09, 95% CI 2.12–30.79) and reoperation due to recidivism (OR 0.1, 95% CI 0.03–0.39), as unfavorable prognostic factors, and that total thyroidectomy for primary operation (OR 8.46, 95% CI 2.21–32.31) was favorable prognostic factor.

**Table 4:** Univariate and multivariate statistical analysis of cancer specific survival

	Univariate			Multivariate		
Factor	*p*	OR	95% CI	*p*	OR	95% CI
Gender (*male* vs. *female*)	0,126	0,42	0,13–1,28			
Age (years) (*<54* vs. *≥55*)	0,056	3,3	0,97–11,26			
Disease duration (years) (*<5* vs. *≥5*)	0,941	1,04	0,34–3,19			
T stadium (*T1 and T2* vs. *T3 and T4*)	0,03	4,18	1,14–15,19			
N stadium (*N0 and Nx* vs. *N1a and N1b*)	0,451	2,19	0,28–16,98			
Tumor type (*minimally* vs. *widely invasive*)	0,684	0,78	0,24–2,54			
Tumor size (mm) (*≤39* vs. *>40*)	0,328	1,99	0,49–7,99			
Capsular invasion (*positive* vs. *no or minimal*)	0,903	1,09	0,29–4,07			
Vascular invasion (*positive* vs. *no or minimal*)	0,149	0,22	0,03–1,71			
Number of tumor focuses (*one* vs. *multicentric*)	0,374	1,72	0,52–5,73			
Affection of both lobes (*yes* vs. *no*)	0,05	3,09	0,97–9,85	0,002	8,09	2,12–30,79
Regional tumor infiltration (*yes* vs. *no*)	0,027	0,28	0,92–0,86			
Primary procedure (*total thyroidectomy* vs. *less than total*)	0,049	3,24	0,99–10,64	0,002	8,46	2,21–32,31
Total thyroidectomy after lobectomy (*yes* vs. *no*)	0,118	0,3	0,68–1,35			
Type of procedure overall (*total thyroidectomy* vs. *less than total*)	0,817	1,17	0,31–4,31			
Reoperation due to relapse (*yes* vs. *no*)	0,001	0,117	0,04–0,35	0,001	0,10	0,03–0,39
Surgeon’s experience (years) (*<10* vs. *10+*)	0,421	0,63	0,2–1,95			
Radioiodine ablation (*yes* vs. *no*)	0,921	1,12	0,1–12,45			
Diabetes mellitus (*yes* vs. *no*)	0,603	21,78	0,001–2,393,431			
Hypertension (*yes* vs. *no*)	0,292	0,53	0,16–1,71			
Other malignancies (*yes* vs. *no*)	0,519	23,03	0,002–315,487			
Thyroglobulin (ng/mL) (*<300* vs. *>300*)	0,695	1,62	0,14–18,38			

**Fig. 4:**
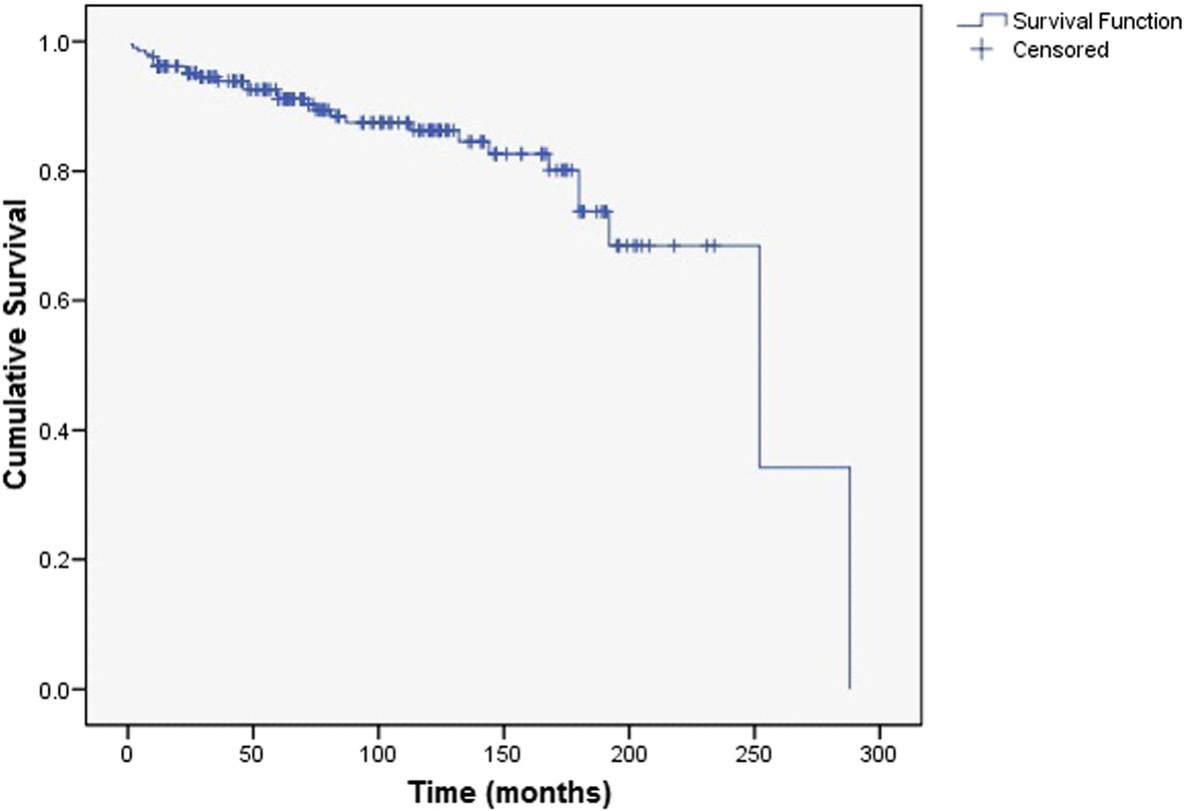
Kaplan-Meier disease free interval curve

In 22 patients re-operation was performed due to local relapse or lymphadenopathy. Another 7 patients died due to the local relapse of the tumor, but since the disease was already in the advanced stage, those patients were not operated. The overall relapse rate was 12.1%. The average time to the relapse was 90.74 ± 85.4 months (range, 1–288 months). The average time without any signs of the disease was 222.4 months (95% CI: 197.8–246.9). One-year disease free interval was 96.1%, five-year was 91.1%, ten-year was 86.2% and twenty-year disease free interval was 68.5% (Fig. 4).

According to the univariate analysis for disease free interval, age, capsular invasion, vascular invasion, surgeon’s experience, ablative radioiodine treatment, hypertension and the presence of other malignancies were associated with shorter survival. Independent predictors for disease free interval were age at diagnosis (OR 1.4, 95% CI 1.01–1.93), capsular invasion (OR 1.59, 95% CI 1.13–2.22), as unfavorable prognostic factors, and surgeon experience (OR 0.62, 95% CI 0.44–0.85) and presence of other malignancies (OR 0.58, 95% CI 0.35–0.96) (Table 5), as favorable prognostic factors.

**Table 5:** Univariate and multivariate statistical analysis of disease free interval

	Univariate			Multivariate		
Factor	*p*	OR	95% CI	*p*	OR	95% CI
Gender (*male* vs. *female*)	0,188	0,79	0,55–1,12			
Age (years) (*<54* vs.* ≥55*)	0,001	1,65	1,22–2,22	0,043	1,4	1,01–1,93
Disease duration (years) (*<5* vs.* ≥5*)	0,073	0,75	0,55–1,02			
T stadium (*T1 and T2* vs. *T3 and T4*)	0,337	0,86	0,64–1,16			
N stadium (*N0 and Nx* vs. *N1a and N1b*)	0,438	0,70	0,29–1,71			
Tumor type (*minimally* vs. *widely invasive*)	0,485	0,89	0,65–1,22			
Tumor size (mm) (*≤39* vs. *>40*)	0,723	1,06	0,77–1,44			
Capsular invasion (*positive* vs. *no or minimal*)	0,001	1,71	1,23–2,37	0,007	1,59	1,13–2,22
Vascular invasion (*positive* vs. *no or minimal*)	0,002	1,64	1,19–2,23			
Number of tumor focuses (*one* vs.* multicentric*)	0,364	0,84	0,58–1,22			
Affection of both lobes (*yes* vs. *no*)	0,123	1,33	0,92–1,9			
Regional tumor infiltration (*yes* vs. *no*)	0,19	1,37	0,86–2,18			
Primary procedure (*total thyroidectomy* vs. *less than total*)	0,335	1,18	0,84–1,63			
Type of procedure overall (*total thyroidectomy* vs. *less than total*)	0,254	1,22	0,86–1,71			
Surgeon’s experience (years) (*<10* vs. *10+*)	0,001	0,58	0,42–0,79	0,003	0,62	0,44–0,85
Radioiodine ablation (*yes* vs. *no*)	0,026	0,69	0,49–0,95			
Diabetes mellitus (*yes* vs. *no*)	0,097	0,59	0,32–1,09			
Hypertension (*yes* vs. *no*)	0,009	0,67	0,5–0,9			
Other malignancies (*yes* vs. *no*)	0,001	0,45	0,27–0,73	0,034	0,58	0,35–0,96
Thyroglobulin (ng/mL) (<*300* vs. >*300*)	0,976	1,01	0,55–1,83			

## Discussion

Since Hurthle cell carcinoma is rare tumor, there is relatively small number of publications regarding this topic; furthermore, these publications are mostly based on the experiences of the individual institutions with a relatively small number of patients during various time intervals [1, 6–8]. Even rarer are the papers that deal with population registers. The three largest series are studies of Nagar et al., Bhattacharyya et al. and Goffredo et al. who processed the registers from the United States [1, 2, 4]. To the best of our knowledge, this is the largest series of Hurthle cell carcinoma patients analyzed and operated at a single center.

The average age of our patients was 54; this is in concordance with other large Hurthle cell carcinoma studies: Petric et al. and Chindris et al. found that an average age at the time of surgery was 62, Sugino et al. 58, Kushchayeva et al. 55.2 and Goffredo et al. 57.6. [2, 6, 9–11] According to the available literature, patients with Hurthle cell carcinoma are older when compared whit patients with other types of thyroid cancers. In a large population study, Goffredo et al. found that Hurthle cell carcinoma occurs almost ten years later than other well-differentiated cancers: 57.6 vs. 48.9 years, respectively [2]. Also, Sugino et al. have found that Hurthle cell carcinoma develops ten years later even if compared to the follicular carcinomas: 58 vs. 48 years, respectively [10]. On the other hand, there are disagreements regarding the age difference between the Hurthle cell adenoma and Hurthle cell carcinoma. Study of Barnabei et al. and one study from our center showed that there was no difference regarding age (49 years), while study of Lopez-Penabad et al. showed that patients with Hurthle cell carcinoma are older at the time of surgery than patients with Hurthle cell adenomas, 51.8 years vs. 43.1 years [12–14].

The average age at the moment of death of the patients with Hurthle cell carcinoma in our series was 64.7 years, and this was more than 10 years less than the average life expectancy in Serbia (74.9 Eurostat Life expectancy at birth) [15]. On the contrary, patients who were operated for Hurthle cell carcinoma and died due to other reasons experienced the average life expectancy. So, based on these results, beside the fact that Hurthle cell carcinoma has low mortality rate, it can also be concluded that patients with aggressive disease and/or inadequately treated patients have shorter life expectancy.

Most of contemporary literature data agrees that the average tumor size at the time of surgery ranges from 25 to 48 mm [2, 4, 6, 13]. In our study, the average tumor size was 41 mm. As the vast majority of endocrine system tumors, Hurthle cell carcinoma also occurs more frequently in females. In our study the female-to-male ratio was 3.6:1, while Mills et al. showed ratio of 1.6:1, Kushchayeva et al. 2.3:1, Nagar et al. 2:1 and Petric et al. 3.2:1 [1, 6, 8, 11].

The surgical procedure of choice in the treatment of Hurthle cell carcinoma should be total thyroidectomy, so that adequate suppressive levothyroxine therapy and ablative radioiodine therapy for stages over T2 could be applied. Still, when procedure less than total thyroidectomy is performed, completion of total thyroidectomy is not always indicated. In our series, in 68.6% of patients a total thyroidectomy was conducted. In other series the percentage of total thyroidectomy ranges from 27.4% to 80% [2, 8, 10, 11, 16]. At our Institution, the completion of thyroidectomy is performed in cases of suspected changes in the contralateral lobe, which tends to increase in spite of suppressive therapy with levothyroxine, or if lesions of the thyroid capsule were present, so ablative radioiodine therapy is indicated. Out of 66 patients who have initially undergone lobectomy, completion of thyroidectomy was performed in 21. Out of those patients, Hurthle cell carcinoma focus was found on the contralateral side in 12 patients (57.1%). In a large series of Sugino et al. among patients who have undergone completion of total thyroidectomy, in 12.8% focus of Hurthle cell carcinoma was found at the contralateral side [10].

In our study locoregional metastases were present in 7.7% of those patients in whom lymph nodes were extirpated during the primary surgery. This percentage is similar in comparison to the one found in a large population study, conducted by Goffredo et al. (5.3%). [2] On the contrary, Sugino et al. found positive lymph nodes in 21.9% of patients, in a large series [10].

The percentage of patients who had a relapse in our study was 12.1%. This percentage is among the lowest, when compared to the other studies that have researched Hurthle cell carcinoma, in which relapse was found in 10.5% to 43% of patients [2, 6, 8]. This can be explained by the good selection of patients, multimodal treatment approach along with an adequate use of radioiodine after total thyroidectomy, and by the fact that total thyroidectomy was performed in a relatively high percentage of patients in our study.

In our study, overall ten-year survival was 77.2%, while cancer-specific survival was 92.5%. Our results and the results from other similar studies regarding the overall survival and cancer-specific survival are shown in Table 6 [1, 2, 4, 6–8, 10, 11, 14, 16, 17]. A ten-year cancer-specific survival in other studies ranges from 49% to 93.1%. There is a great number of factors that affect the disease specific survival, for example: in studies that were conducted in tertiary institutions patients are more likely to be in the advanced stages, so disease specific survival is shortened. Similarly, studies that include earlier periods, from earlier years, have lower percentages of survival. Thus, studies of Mills et al., Stojadinovic et al. and Lopez-Penabad et al., which include periods from ‘40-ies report disease-specific survival rates in a range of 64% to 73%, while studies involving patients from the ‘70s and ‘80-ies report a significantly higher percentages, up to 90%. The reason for better disease-specific survival rates in later years most likely lies in the fact that nowadays, tumors are revealed at an earlier stage, that less radical surgical procedures, such as bilateral subtotal lobectomy and Dunhill’s procedure, were applied more at that time and that radioiodine was less frequently used. Besides, disease-specific survival rate is improved for other types of well-differentiated thyroid cancers: the study of Chow et al. showed improvement in survival rate in years between 1960 and 2000, in 1.348 patients with differentiated thyroid cancer [18]. However, the study of Goffredo et al. showed that the trend has stabilized in the last two decades, so Hurthle cell carcinoma’s survival does not change, unlike other differentiated thyroid cancers [2]. Similar findings were presented by Roman et al. in a study that examined the survival of patients with medullary carcinoma: survival rates did not show significant changes during the period from 1973 to 2002 [19].

**Table 6:** Ten years survival in patients with Hurthle cell carcinoma

	Period of study	Number of patients	Overall survival (%)	Cancer specific survival (%)	Disease free interval (%)
Haigh et al. (SEER) [16]	1988–1993	172	73		
Lopez-Penabad et al. [14]	1944–1995	89	55	64	
Bhattacharyya et al. (SEER) [4]	1973–1998	555	71.1		
Stojadinovic et al. [7]	1940–2000	56		73	61
Kushchayeva et al. [11]	1976–2002	33		49	40.5
Mills et al. [8]	1946–2003	62		64	43
Jillard et al. (National Cancer Database) [17]	1998–2006	1909	70.7		
Nagar et al. (SEER-9) [1]	1975–2009	1416	77.1		
Goffredo et al. (SEER) [2]	1988–2009	3311		91	
Sugino et al. [10]	1989–2010	73		93.1	
Petric et al. [6]	1972–2011	108		88	68
Our study	1995–2014	239	77.2	92.5	86.2

*SEER* surveillance, epidemiology, and end results

In present study, independent predictive factors for shorter overall survival were age over 55, T3 and T4 stadium, alterations in both thyroid lobes and the need for re-operation due to local relapse. When cancer specific survival was observed, multivariate regression analysis showed that affection of both thyroid lobes and need for reoperation due to local relapse were unfavorable prognostic factors and that total thyroidectomy as primary procedure was independent favorable predictor. Recent study conducted by Petric et al. showed that independent prognostic factors for cancer specific survival were age, distant metastases and residual tumor after surgery [6]. Bhattacharyya et al. found that age at the time of diagnosis, male gender and increasing tumor size were statistically significantly associated with shorter cancer specific survival in multivariate analysis [4]. Results of a large, population-based study that included 3311 patients found that age at diagnosis over 45, marital status, tumor size ≥4 cm and extrathyroidal invasion were independently associated with lower cancer specific survival. Also, those authors showed that patients who were not surgically treated had worse prognosis [2]. Based on the results of other studies, independent negative predictors for cancer specific survival are older age, tumor size >4 cm, positive extrathyroid invasion, higher T stadium, capsular invasion, less extensive surgery and the presence of more than one focuses, residual tumor tissue following surgery and regional and/or distant metastases [7, 8, 14].

In our study, a ten-year disease free interval was 86.2%. In the available literature there are insufficient data, so a ten-year disease free interval varies in a range from 40.5 to 68% (Table 6) [6–8, 11]. Likewise, only few studies investigated prognostic factors for disease free interval, particularly – independent predictors. Petric et al. found that male gender, age over 45, regional metastasis at the moment of primary surgery and residual tumor after surgery were associated with unfavorable prognosis [6]. Stajadinovic et al., analyzed 60 years of experience in the treatment of patients with Hurthle cell carcinoma and found that extrathyroidal invasion and lymphonodal metastases were associated with shorter disease free interval [20]. Results from our study showed that age over 55, positive capsular invasion, surgeon’s experience and the presence of non-thyroid malignancies were predictors of shorter disease free interval.

The fact that the study was conducted in a single center, that the data are uniform and that all patients were operated with the same doctrine, should be considered as advantages of the current study.

Our study has several limitations. The main limitation certainly represents retrospective design; also, this study is observational and not randomized. Furthermore, there is a relatively small number of subjects. We believe that it would be useful to perform multicentric study with a higher number of patients.

## Conclusions

Hurthle cell carcinoma is rare tumor with an encouraging prognosis. After adequate surgical treatment, relapses are rare. A ten-year cancer specific survival is 92% and even 86% of patients have no signs of relapse ten years after surgery.

## Abbreviations

CI: Confidence interval
OR: Odds ratio
SD: Standard deviation
TNM: Tumor Nodes Metastasis
